# Punch and Judy in the City Road

**Published:** 1914-01-03

**Authors:** 


### CHRISTMAS IN THE HOSPITALS.

### Punch and Judy in the City Road.

On Saturday, December 20, at the Royal Hospital for Diseases of the Chest, City Road, nearly two hundred children spent a happy evening. In the new out-patient department a splendid entertainment was provided, after a bounteous supply of tea and cake had been absorbed. An enormous Christmas-tree gaily decorated with electric lights fairly groaned under its burden of presents for everyone there. The wistful glances of the poor children of Shoreditch and the vicinity were soon changed into shouts of glee as the little recipients clutched their toys and bags of sweets from the hands of the burly Santa Claus. This benevolent being, in the person of Dr. McNaboe, made his dramatic entry in the true and approved style by climbing through the window. He was accompanied by a most realistic teddy-bear (Mr. Hamilton, brother of the resident medical officer), who was a source of great joy throughout the evening. The unqualified success of the entertainment may be justly credited to the efforts of the matron, Miss Rundle, and the two out-patient sisters, Miss Hayes and Miss Gaudie. These last two ladies worked hard and long to bring a few hours of gladness to their charges, for all the guests were drawn from the out-patient practice of the hospital.

Apart from the ceremony of stripping the Christmas-tree of its presents, an immense interest was shown by this juvenile audience in the Punch and Judy. It was little short of a revelation to see how keenly excited the children were over the doings of Punch, and how consistently they were all for the dog Toby in his moments of maltreatment. The children of Shoreditch cannot be said to be so spoilt by picture palaces and ragtime but that they appreciated this ancient joy; it must be confessed that the grown-ups, too, thoroughly enjoyed it. Among others present were several members of the staff: Dr. Phear, Dr. Davies, Dr. Symes-Thompson, Dr. Stott, the newly-appointed cardiologist, and a few others.

